# Everybody's Page

**Published:** 1890-05-24

**Authors:** 


### Everybody's Page.

The last cure for insomnia does not commend itself to us altogether, viz., raw onions to be eaten before bed-time!

Russian ladies are doing excellent service as doctors in Asiatic Russia among the Persians, Afghans, and others. Their services among the women and children are shown by the returns to be highly appreciated.

A sufferer says "oil of wintergreen" is a very good thing to relieve the pain of rheumatism. There appear to be as many "marvellous cures" for rheumatism as there are sufferers from it, but it would seem that the "specific remedy" has yet to be discovered.

Helmholtz is one of the men of science who through illness found his vocation. Being laid up all one autumn with typhoid fever, he was tended free of cost in the hospital of which he was a pupil. With the money thus saved he bought a microscope and first entered on the study of science.

Smoking is an illustration of how easy it is to suffer for another man's sins. Spanish ladies suffer considerably from irritation of the throat caused not by the habit of smoking themselves, but by the constant fumes of tobacco which is indulged in so freely by their male relatives. Sir Morell Mackenzie warns all vocalists against smoking concerts, smoking carriages, or any other place where the atmosphere is laden with tobacco.

Cold is the elementary cause which throws some animals into a somnolent state during the winter, making them insensible to the ordinary conditions of life, and reducing the ordinary heart pulsations and respiration in a very marked degree. Some of us will remember seeing this well illustrated by dormice and squirrels. Spallanzani is said to have suspended successfully the vitality of frogs for two years, by preserving them in snow. At the end of this time they were quite dry and stiff. Gradually increasing heat, however, restored them to their customary sprightliness.

At the annual meeting of the German Surgical Congress, held at Berlin last month, Professor Ponfick, of Breslau, discussed the possibility of successfully removing large portions of the liver. Experiments on rabbits led him to discover that even after the removal of three-quarters of the liver, many of the animals, though at first greatly prostrated, not only survived for a short time, but completely recovered. On hearing of this our heart leapt for joy at the vision of life without a liver, but, alas! our hopes are dashed to the ground, for the professor finds it impossible to remove the whole liver with success.

The Concord Prison in Massachusetts sounds to us a veritable paradise. The inmates, by good behaviour, can obtain release in eleven months, and the maximum detention is five years, not to mention that intellectual study is the order of the day there. Surely it would subdue the most rampant convict to be set down to describe "rectilinear perspective," or to describe what is meant by division of labour and what is its result, or to give the "percentage of words in the dictionary of Anglo-Saxon origin." This is very high-class no doubt, but somehow it comes as a relief to our feelings to learn that such things as stucco work, farm work, printing, &c., are also thought necessary to the prisoners' education.

Kola-nut seems to be a worthy rival of chocolate for the purpose of sustaining bodily vigour. According to the *New York Medical Record*, experiments with it in the German army have met with the greatest success.

We are always hearing of the bad influence Europeans have on native races as regards drink. Zulus, however, says Sir C. Mitchell, are proof against bad example, and though they mix very freely with Europeans keep up an excellent reputation for sobriety.

Dangers certainly lurk in unexpected places, and very soon we shall be solemnly asking one another, "What can we safely eat?" Even celery has turned traitor, and has been discovered by Dr. Cresson, of Philadelphia, to contain typhoid germs. What a heartrending discovery—at least, it appeals as such to our feelings.

A common error is to suppose that the deeper the surgeon's knife goes the greater the pain. As a matter of fact, the flesh and inner organs are without sensation while healthy, and the skin is, as Sir John Lubbock puts it, the sentinel which by its sensitiveness to pain warns the vital parts of approaching danger. Pain would be without purpose if deeply seated.

It is the fashion or spirit of the age to cry down all sorts of restraint of whatsoever kind, to reject the wisdom of the elders, and to cry loudly for independence. Excellent as is a love of freedom, it strikes us that there is a sort of pseudo-independence running riot which does infinite harm in all classes. Love of self-help and industry is not the key-note of this latter kind; love of self-will is more like it, a general "don't care" to every moderate piece of advice offered.

The rules which have been issued recently by the Government of India for the regulation of the marriage expenses of the Kadya Kanbi caste in the Bombay Presidency take us back to the sumptuary laws of this country in the middle ages. Even the costermonger of Whitechapel would, in these days of freedom and independence, revolt against a law which forbade him to spend more than one shilling or the value of two or three cocoanuts on a wedding gift for his bride-elect.

The thirst for gain will break down most barriers; nothing is sacred to it. Years ago the hillmen of the Caucasus had a deep respect for the burial places of their ancestors; now they have found out that these said ancestors were foolish enough to have all sorts of valuable things buried with them, so exit respect and enter greed, and the forefathers are dug up. The hillmen find archæological remains have a good sale, but while they grab at the gold or silver finds, they unfortunately do not appreciate stone arrows or hatchets, and throw them away. It makes us wish the Caucasus were a little nearer home.

"No gratuities allowed" is a prominent notice seen on the walls of the Aerated Bread Company's shops. And yet often we wish we were allowed to show some small sign of material gratitude to the willing girl who brings us our welcome cup of tea. Perhaps some of our readers have not noticed the boxes on the bar, with "Provident Fund" printed on them. Our pennies can go into them, and be of real use. By-the-bye, we wish one or two of the down-stair rooms of these well-managed places could be better ventilated—they are very stuffy, and this detracts from one's enjoyment of a roll and a glass of milk very considerably.

